# A novel multidimensional uncalibration method applied to six-axis manipulators

**DOI:** 10.3389/fnins.2023.1221740

**Published:** 2023-07-14

**Authors:** Haitao Qiu, Dan Huang, Bo Zhang, Ming Wang

**Affiliations:** ^1^School of Electric Power Engineering, South China University of Technology, Guangzhou, China; ^2^School of Mechanical and Automotive Engineering, South China University of Technology, Guangzhou, China; ^3^School of Mechanical Engineering, Ningxia University, Yinchuan, China

**Keywords:** image Jacobian matrix, machine vision, uncalibrated visual servo, dynamic quasi-Newton algorithm, robot

## Abstract

This study proposes a multidimensional uncalibrated technique for tracking and grasping dynamic targets by a robotic arm in the eye-in-hand mode. This method avoids complex and cumbersome calibration processes, enabling machine vision tasks to be adaptively applied in a variety of complex environments, which solved the problem of traditional calibration methods being unstable in complex environments. The specific method used in this study is first, in the eye-in-hand mode, the robotic arm moves along the *x, y*, and *z* axes in sequence, and images are taken before and after each movement. Thereafter, the image Jacobian matrix is calculated from the three (or more) sets of images collected. Finally, the robotic arm converts the target coordinates in the real-time captured images by the camera into coordinates in the robotic arm coordinate system through the image Jacobian matrix and performs real-time tracking. This study tests the dynamic quasi-Newton method for estimating the Jacobian matrix and optimizes the initialization coupling problem using the orthogonal moving method. This optimization scheme significantly shortens the iteration process, making the uncalibrated technology more fully applied in the field of dynamic object tracking. In addition, this study proposes a servo control algorithm with predictive compensation to mitigate or even eliminate the systematic error caused by time delay in dynamic target tracking in robot visual servo systems.

## 1. Introduction

In the 1960s, due to the development of robotics and computer technology, people began to study robots with visual functions, and in the 1980s, the concept of robot visual servo was proposed. In the following decades, robot visual servoing underwent rapid development. Visual servo control mainly inputs visual information provided by visual sensors into the control system, enabling the control system to process external information. Traditional robot visual servo systems are mostly implemented based on system model calibration technology (Gans, 2003; Huang et al., 2022), which mainly involves models such as camera models, robot models, and target object models. The camera model refers to the internal and external parameters of the camera; the robot model generally refers to the robot kinematics model; the target model mainly refers to the depth information from the target to the end of the robotic arm, as well as the pose and motion parameters of the target in a fixed coordinate system. In the traditional robot visual servo system, the first step is to complete the calibration of the camera and the calibration between the camera and the robot (Hutchinson et al., 1996) to obtain an accurate conversion matrix between the image coordinate system and the robot coordinate system. Then, based on the calibrated transformation matrix, the coordinates of the target object in the image captured by the visual system are converted to obtain the pose of the robot in the coordinate system. Finally, the robot tracks, locates, and grasps the target object in the camera's field of view based on the converted coordinate information (Kang et al., 2020). Throughout the entire work process, the accuracy of the transformation matrix between the image coordinate system and the robot coordinate system is heavily dependent (Malis, 2004). The calibration work between the camera and the robot is extremely cumbersome, requiring data such as the internal and external parameters of the camera, the motion model of the robot model, and the position relationship between the camera and the fixed position of the robot. However, in practical applications, replacing the camera or camera lens, or loosening the installation position between the camera and the robot can cause deviation in the calibration results, requiring complex calibration work to be carried out again. The traditional calibration methods for robot visual servo systems make it difficult for them to operate in complex working environments, which is currently a bottleneck limiting the development of robot visual servo systems.

To break the bottleneck, researchers have begun to focus on studying the “eye-in-hand” structure visual servo control method for calculating the image Jacobian matrix without knowing system parameters. The robot visual servo system still needs to overcome many technical difficulties to be put into normal use in various complex production environments.

The development of uncalibrated technology between cameras and robots without knowing system parameters can be divided into multiple stages: 1. The robot visual servo system achieves precise positioning and grasping of static targets through uncalibrated technology; 2. the robot visual servo system achieves tracking and positioning of dynamic targets through uncalibrated technology; and 3. the robot visual servo system achieves practical production applications with low latency and high accuracy in complex environments.

The fundamental goal of implementing a robot visual servo system is to achieve precise positioning and grasping of static targets. Hosoda and Asada first proposed the exponential weighted recursive least squares method to obtain the Jacobian matrix. This method achieves servo tracking and positioning of stationary targets in an uncalibrated state, but there are still shortcomings in terms of system stability and accuracy of image feature extraction (Hosoda and Asada, 1994; Cao et al., 2022a,b). Yoshimi and Allen introduced an additional robotic arm to explore motion and observed corresponding changes in image features during each calculation cycle. Then, they combined the least square method to calculate the Jacobian matrix of the current image, achieving more accurate two-dimensional target tracking. However, this method is too cumbersome and lacks real-time performance, making it difficult to apply in practical work (Yoshimi and Allen, 1995). In addition, many researchers have obtained the image Jacobian matrix by converting the online estimation of the Jacobian matrix into system state observation (Jianbo, 2004) or recursive formula calculation (Longjiang et al., 2003) and tested the algorithm from four aspects: initial value, operating range, stability, and robustness. Simulation experiments have been conducted to verify the reliability of the algorithm (Hao and Sun, 2007). At this stage, it is possible to use robot visual servo systems for positioning and grasping static targets in industrial production applications that meet various requirements (Singh et al., 1998). Compared to traditional calibration methods (Jingmei et al., 2014), it avoids the tedious process of repeated calibration.

With the development of production technology, the function of only achieving precise positioning and grasping static targets no longer meets the production needs of enterprises. Therefore, Piepmeier proposed the Broyden method to estimate the image Jacobian matrix, thereby achieving tracking and positioning of moving targets. However, when the deviation of image features is large, the performance of the control system will decrease, even leading to control failure (Piepmeier and Lipkin, 2003). When the robot visual servo system tracks irregularly moving targets (Haifeng et al., 2010), it is necessary to improve the real-time performance of the system (Zaien et al., 2014) and the convergence speed of the image Jacobian matrix (Chang et al., 2020). However, while ensuring the real-time performance of the system, it can also lead to problems such as slow recognition speed and low accuracy of the visual system during high-speed movement. Many researchers have combined BP neural networks and genetic algorithms (Samad and Haq, 2016; Chen et al., 2020; Yuhan et al., 2021; Wu et al., 2022) and applied them to real-time image processing in the visual system, improving the processing speed of the visual system, improving the processing speed of the visual system. In addition, it is necessary to improve the robustness of the robot's visual servo system (Li et al., 2009; Hao et al., 2020) to adapt to stable operation in various complex environments. For example, in the field of medical equipment, the robot servo system needs to operate absolutely accurately and stably (Piepmeier, 2003; Gu et al., 2018; Zhang et al., 2020), thus improving the robustness and anti-interference of the system is very important (Cao et al., 2021; Gao and Xiao, 2021).

This study researches the application background of tracking and trajectory coverage of irregular dynamic targets. First, an online estimation test of the dynamic quasi-Newtonian Jacobian matrix was conducted in the simulation system. After analyzing the simulation test results, the system initialization process was targeted and optimized, significantly improving the convergence speed of Jacobian matrix iteration. In addition, this study also proposes a predictive compensation Jacobian matrix PI control algorithm to solve the lag problem of the visual system in the dynamic tracking process, effectively improving the accuracy of the robot servo system in the dynamic tracking process.

The remainder of this article is structured as follows. In Section 2, a detailed introduction is given to the control system. This includes the hardware composition of the control system, theoretical deduction of uncalibrated technology, and an introduction to servo control algorithms. In Section 3, we present the experimental results and discuss them. These results include the iterative process for the proposed uncalibrated visual servo system and the optimized iterative process. In addition, a comparative analysis of the research and experimental data conducted in this study is also presented in Section 4.

## 2. Control system

### 2.1. Operating platform

**Figure 1:**
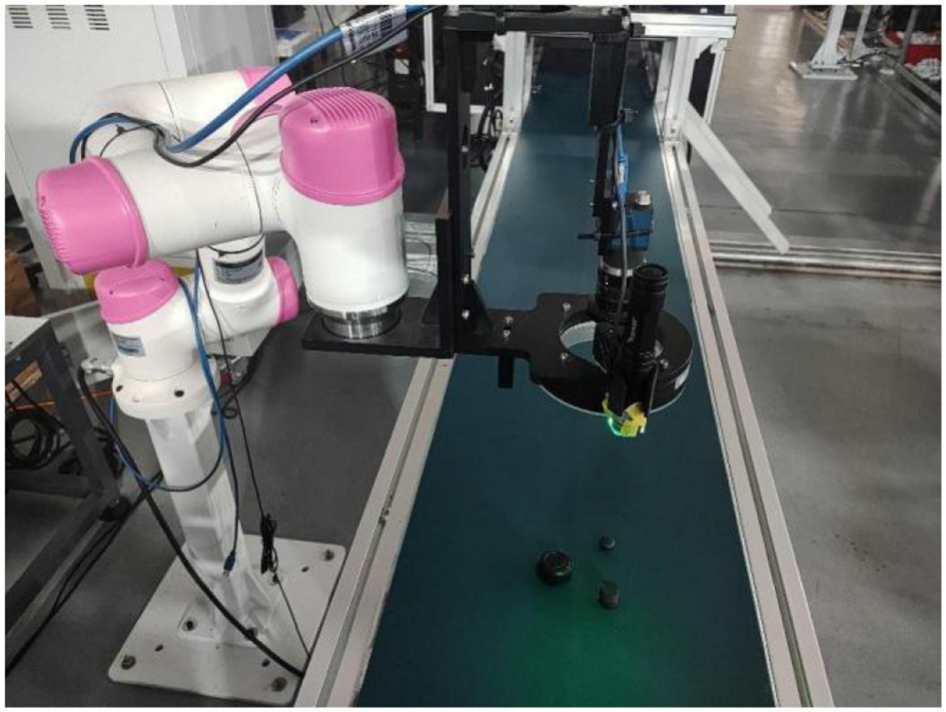
Bozhilin 6-axis robotic arm platform.

**Figure 2:**
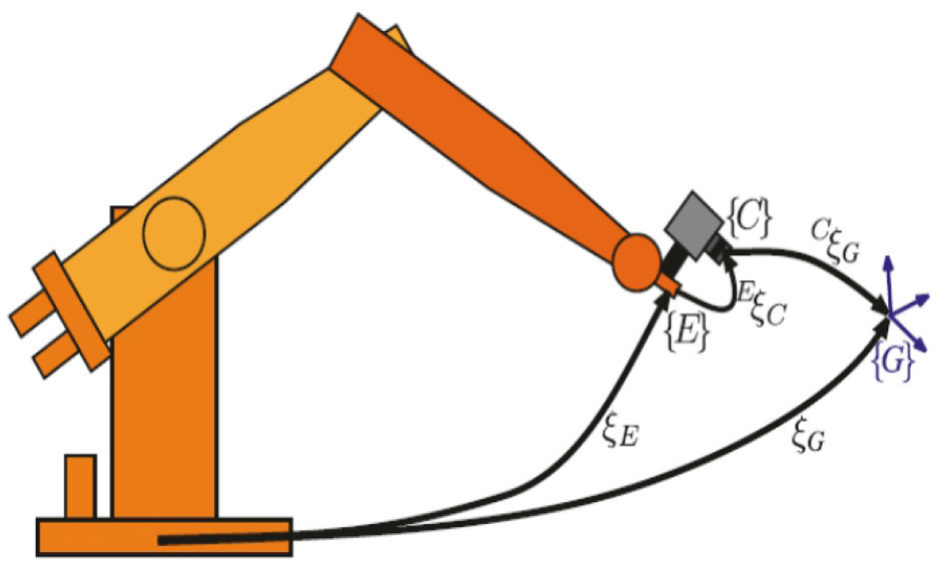
Schematic diagram of the eye-in-hand model.

The robot uncalibrated servo technology reviewed in this study is based on the application of tracking and coating trajectories to moving targets. The technology analyzed in this study can be applied to different fields such as the application of mobile robots to building cracks and robot welding. The robot platform used in this study is a six-axis industrial robot independently developed by Bozhilin, as shown in Figure 1. A Daheng high-speed industrial camera is installed at the end of the robotic arm to collect image information within the working range of the robotic arm. The camera used needs to have a large field of view, as the target object cannot leave the camera's field of view during uncalibrated initialization; otherwise, it will cause the Jacobian matrix error to increase. The camera and robot are installed in the eye-in-hand mode, and the model diagram is shown in Figure 2.

### 2.2. Process of uncalibration

**Figure 3:**
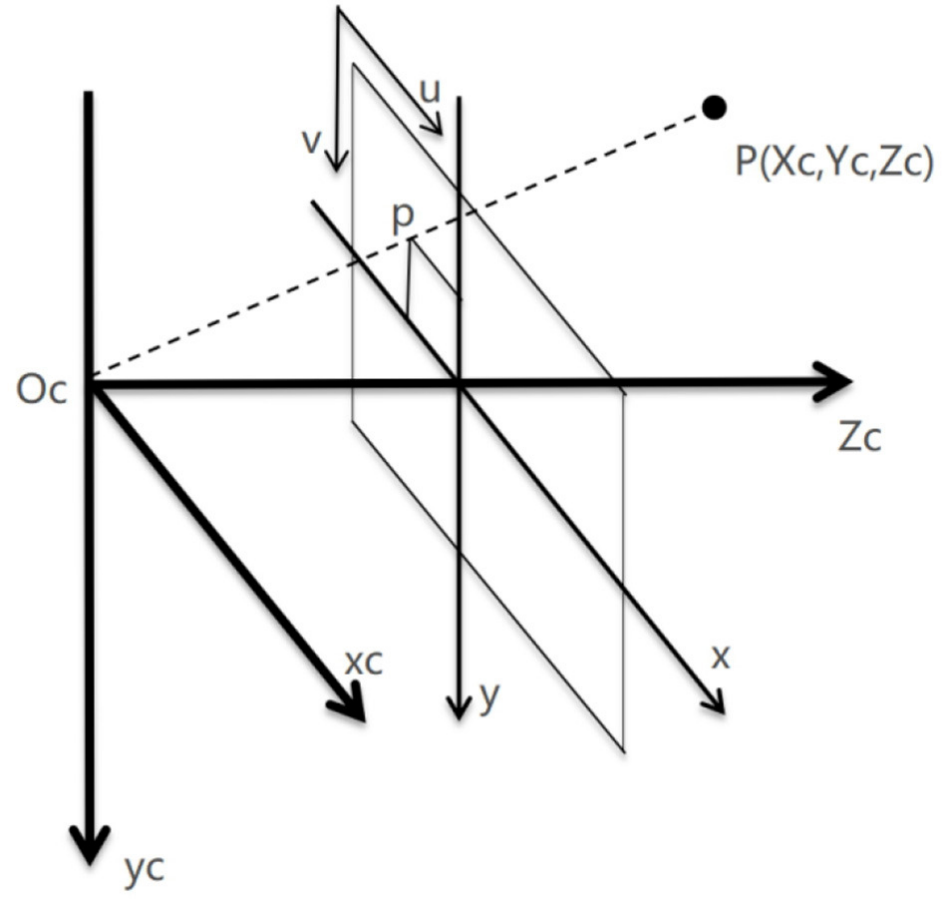
Camera pinhole imaging model.

Uncalibration technology, such as traditional calibration techniques, is used to describe the relationship between the speed of robot end effectors and the rate of feature change in the image. Assuming a point P in three-dimensional space, based on the traditional camera pinhole imaging model as shown in Figure 3, it can be concluded that


(1) $$\begin{array}{l} \{\begin{array}{c} x_{i}=\frac{f}{z_{c}}x_{c}\\ y_{i}=\frac{f}{z_{c}}y_{c} \end{array} \end{array}$$

*P*_*c*_(*x*_*c*_, *y*_*c*_, *z*_*c*_) is the coordinate of point *P* in the camera coordinate system, *P*_*w*_(*x*_*w*_, *y*_*w*_, *z*_*w*_) is the Cartesian coordinate of point *P* in the world coordinate system (robotic arm base coordinate system), *P*_*I*_(*x*_*i*_, *y*_*i*_) is the projection coordinate of point *P* in the camera plane coordinate system, and (*u*_*i*_, *v*_*i*_) is the pixel coordinate in the pixel plane coordinate system.

The relationship between the camera imaging plane coordinate *P*_*I*_(*x*_*i*_, *y*_*i*_) and the pixel plane coordinate (*u*_*i*_, *v*_*i*_) is


(2) $$\begin{array}{l} \{\begin{array}{c} u_{i}=\frac{x_{i}}{dx}+u_{0}\\ v_{i}=\frac{y_{i}}{dy}+v_{0} \end{array} \end{array}$$

In the above equation, *u*_0_ and *v*_0_ are the pixel coordinates of the penetration point of the camera's optical axis in the pixel plane, while *dx* and *dy* represent the spatial distance represented by a single pixel in the *X* and *Y* directions in the pixel plane, respectively.

Convert the above equation into a matrix equation as follows:


(3) $$\begin{array}{l} \left[\begin{array}{c} u_{i}\\ v_{i}\\ 1 \end{array}\right]=\left[\begin{array}{c} \begin{array}{ccc} \frac{1}{dx} & 0 & u_{0} \end{array}\\ \begin{array}{ccc} 0 & \frac{1}{dy} & v_{0} \end{array}\\ \begin{array}{ccc} 0 & 0 & 1 \end{array} \end{array}\right]\;[\begin{array}{c} x_{i}\\ y_{i}\\ 1 \end{array}] \end{array}$$

Assuming that the focal length of the camera is *f* , under the ideal pinhole model of the eye-in-hand system, the conversion relationship between the camera coordinate system and the pixel coordinate system is


(4) $$\begin{array}{l} \left[\begin{array}{c} u_{i}\\ v_{i} \end{array}\right]=\frac{f}{z_{c}}\left[\begin{array}{c} x_{c}\\ y_{c} \end{array}\right] \end{array}$$

According to the motion equation of the robot's end effector, we have


(5) $$\begin{array}{l} P^{c}={\text{Ω}}^{c}*P^{c}+T^{c} \end{array}$$

(6) $$\begin{array}{l} \{\begin{array}{c} x_{c}=z_{c}w_{y}+T_{x}-\frac{v_{i}z_{c}}{f}w_{z}\\ y_{c}=\frac{u_{i}z_{c}}{f}w_{z}-z_{c}w_{x}+T_{y}\\ z_{c}=\frac{v_{i}zc}{f}w_{x}-\frac{u_{i}z_{c}}{f}w_{y}+T_{z} \end{array} \end{array}$$

Converting the above equation into a matrix equation, we obtain as follows:


(7) $$\begin{array}{l} \left[\begin{array}{c} u\\ v \end{array}\right]=\left[\begin{array}{cccccc} \frac{f}{z_{c}} & 0 & -\frac{u_{i}}{z_{c}} & -\frac{u_{i}v_{i}}{f} & \frac{f^{2}+u_{i}^{2}}{f} & -v_{i}\\ 0 & -\frac{f}{z_{c}} & -\frac{v_{i}}{z_{c}} & -\frac{f^{2}+V_{i}^{2}}{f} & \frac{u_{i}v_{i}}{f} & u_{i} \end{array}\right]\cdot \left[\begin{array}{c} T^{c}\\ {\text{Ω}}^{c} \end{array}\right] \end{array}$$

In practical applications, it is impossible to obtain the transformation matrix between (*u*_*i*_, *v*_*i*_) and [*T*^*c*^, Ω^*c*^]*T* by measuring each variable in the above equation. Therefore, the variables in the matrix are considered unknown:


(8) $$\begin{array}{l} \left[\begin{array}{c} u\\ v \end{array}\right]=\left[\begin{array}{cccccc} a_{11} & a_{12} & a_{13} & a_{14} & a_{15} & a_{16}\\ b_{11} & b_{12} & b_{13} & b_{14} & b_{15} & b_{16} \end{array}\right]\cdot \left[\begin{array}{c} T_{x}\\ T_{y}\\ T_{z}\\ \omega_{x}\\ \omega_{y}\\ \omega_{z} \end{array}\right] \end{array}$$

On the six-axis robotic arm platform, a single feature pixel does not meet the dimensional requirements, so three feature points are taken and stacked up and down:


(9) $$\begin{array}{l} \left[\begin{array}{c} \overset{.}{u_{1}}\\ \overset{.}{\begin{array}{c} v_{1}\\ \overset{.}{u_{2}}\\ \overset{.}{v_{2}}\\ \overset{.}{u_{3}}\\ \overset{.}{v_{3}} \end{array}} \end{array}\right]=\left[\begin{array}{c} \begin{array}{cccccc} a_{11} & a_{12} & a_{13} & a_{14} & a_{15} & a_{16}\\ b_{11} & b_{12} & b_{13} & b_{14} & b_{15} & b_{16} \end{array}\\ \begin{array}{cccccc} a_{21} & a_{22} & a_{23} & a_{24} & a_{25} & a_{26}\\ b_{21} & b_{22} & b_{23} & b_{24} & b_{25} & b_{26} \end{array}\\ \begin{array}{cccccc} a_{31} & a_{32} & a_{33} & a_{34} & a_{35} & a_{36}\\ b_{31} & b_{32} & b_{33} & b_{34} & b_{35} & b_{36} \end{array} \end{array}\right]\cdot \left[\begin{array}{c} T_{x}\\ T_{y}\\ T_{z}\\ \omega_{x}\\ \omega_{y}\\ \omega_{z} \end{array}\right] \end{array}$$

Ḟ represents the rate of change of image features, *J*_0_ represents the Jacobian transformation matrix, and Ṗ represents the motion vector of the robotic arm end effector. The above equation can be expressed as follows:


(10) $$\begin{array}{l} \overset{.}{F}=J_{0}\overset{.}{P} \end{array}$$

In practical applications, we need to convert the two change rates $\overset{.}{F}$ of image features to obtain the motion vector $\overset{.}{P}$ of the robotic arm end effector, so we need to inverse the Jacobian matrix $J={J_{0}}^{-1}$.


(11) $$\begin{array}{l} \overset{.}{P}=J_{0}\overset{.}{F} \end{array}$$

In application, two change rates of image features are obtained from two adjacent images, so discretization of equations is also required. In the process of high-frequency camera image retrieval, we assume that the Jacobian matrix of adjacent two frames of images remains approximately unchanged. The discrete equation can be obtained as follows:


(12) $$\begin{array}{l} F_{\left(n+1\right)}\approx F_{\left(n\right)}+J_{\left(n\right)}\cdot \Delta P_{\left(n\right)} \end{array}$$

(13) $$\begin{array}{l} P_{\left(n+1\right)}\approx P_{\left(n\right)}+{J_{\left(n\right)}}^{\text{-1}}\cdot \Delta F_{\left(n\right)} \end{array}$$

(14) $$\begin{array}{l} J_{}=\Delta F_{}\cdot \Delta {P_{}}^{\text{-1}} \end{array}$$

During the initialization process of the robot visual servo system, there is a coupling relationship between multiple movements of the robot, which can lead to the irreversibility and solvability of the Jacobian matrix. In order to obtain a more accurate Jacobian matrix, this article optimized the initialization process of the robot visual servo system. Therefore, by standardizing the movement direction of the robotic arm during the initialization process, the obtained feature point set is naturally linearly uncorrelated by decomposing the movement of the robotic arm into independent movements of each degree of freedom $\left[\begin{array}{cccccc} T_{x} & T_{y} & T_{z} & \omega_{x} & \omega_{y} & \omega_{z} \end{array}\right]$ during the initialization process. When moving in the independent *T*_*x*_ direction, we get


(15) $$\begin{array}{l} \left[\begin{array}{c} \overset{.}{u_{1}}\\ \overset{.}{v_{1}}\\ \overset{.}{u_{2}}\\ \overset{.}{v_{2}}\\ \overset{.}{u_{3}}\\ \overset{.}{v_{3}} \end{array}\right]=\left[\begin{array}{cccccc} a_{11} & a_{12} & a_{13} & a_{14} & a_{15} & a_{16}\\ b_{11} & b_{12} & b_{13} & b_{14} & b_{15} & b_{16}\\ a_{21} & a_{22} & a_{23} & a_{24} & a_{25} & a_{26}\\ b_{21} & b_{22} & b_{23} & b_{24} & b_{25} & b_{26}\\ a_{31} & a_{32} & a_{33} & a_{34} & a_{35} & a_{36}\\ b_{31} & b_{32} & b_{33} & b_{34} & b_{35} & b_{36} \end{array}\right].\left[\begin{array}{c} T_{x}\\ 0\\ 0\\ 0\\ 0\\ 0 \end{array}\right] \end{array}$$

(16) $$\begin{array}{l} \overset{.}{F}=T_{x}\left[\begin{array}{c} a_{11}\\ b_{11}\\ a_{21}\\ b_{21}\\ a_{31}\\ b_{31} \end{array}\right]+0*\left[\begin{array}{c} a_{12}\\ b_{12}\\ a_{22}\\ b_{22}\\ a_{32}\\ b_{32} \end{array}\right]+0*\left[\begin{array}{c} a_{13}\\ b_{13}\\ a_{23}\\ b_{23}\\ a_{33}\\ b_{33} \end{array}\right]\\ +0*\left[\begin{array}{c} a_{14}\\ b_{14}\\ a_{24}\\ b_{24}\\ a_{34}\\ b_{34} \end{array}\right]+0*\left[\begin{array}{c} a_{15}\\ b_{15}\\ a_{25}\\ b_{25}\\ a_{35}\\ b_{35} \end{array}\right]+0*\left[\begin{array}{c} a_{16}\\ b_{16}\\ a_{26}\\ b_{26}\\ a_{36}\\ b_{36} \end{array}\right] \end{array}$$

After completing the initialization of the image Jacobian matrix, it is necessary to update and iterate the matrix in real time to ensure accuracy during the robot operation process. In the image plane, the difference between the actual feature and the expected feature is *f*(θ, *t*) = *y*(θ, *t*)−y^*^, where θ is the joint angle and *t* is time. Taylor expansion is performed on the deviation function *f*(θ, *t*) and the radiation model is defined as *m*(θ, *t*).


(17) $$\begin{array}{l} m\left(\theta ,t\right)=f\left(\theta_{k},t_{k}\right)+J\left(\theta -\theta_{k}\right)+\frac{\partial f_{k}}{\partial t}\left(t-t_{k}\right) \end{array}$$

At moment *k-1*, we get


(18) $$\begin{array}{l} f(\theta_{k-1},t_{k-1})=m(\theta_{k-1},t_{k-1})\\ =f(\theta_{k},t_{k})+J_{k}(\theta_{k-1}-\theta_{k})+\frac{\partial f_{k}}{\partial t}(t_{k-1}-t_{k}) \end{array}$$

The iterative equation can be obtained as follows:


(19) $$\begin{array}{l} J_{k}=J_{k-1}+\frac{\left(\Delta f_{k}-J_{k-1}\Delta \theta -\frac{\partial f_{k}}{\partial t}\Delta t\right)\Delta \theta^{t}}{\Delta \theta^{t}\Delta \theta } \end{array}$$

### 2.3. Servo control algorithm

The process of running a robot visual servo control system is as follows: first, the visual system captures images and processes them, and then the processed image information inputs into the robot controller to start the robot moving. There is a time delay between the visual system capturing images and the robot starting to move, which can cause systematic errors in the robot's tracking of dynamic targets. Therefore, in the process of robot motion control, this study designs a Jacobian matrix PI control algorithm with predictive compensation to reduce systematic errors caused by the time lag.

Assuming that the expected image feature of the moving target is f^*^(u^*^, v^*^) and the actual feature of the robot pose after the image Jacobian matrix transformation is *f*_*t*_(*u*_*t*_, *v*_*t*_) the actual pose and expected pose feature error of the system are as follows:


(20) $$\begin{array}{l} e\left(t\right)=f^{*}-f_{t} \end{array}$$

In order to improve the real-time performance of the system and ensure that the target motion speed is fast and can complete effective tracking tasks, a predictive compensation method is introduced into the Jacobian matrix control algorithm on the inverse Jacobian matrix visual servo control algorithm, and a Jacobian matrix PI control algorithm with predictive compensation is designed. We define the system image feature error as follows:


(21) $$\begin{array}{l} e_{h}\left(t\right)=f^{d}-{f^{h}}_{t} \end{array}$$

In the above equation, ${f^{h}}_{t}$ is the current image feature, and *f*^*d*^ is the expected image feature. The predicted compensation amount ξ is defined as follows:


(22) $$\begin{array}{l} \xi =kV_{image} \end{array}$$

*V*_*image*_ is the rate of change in image features, and k is the compensation coefficient.

In the process of dynamic target tracking, in order to reduce system tracking error, the PI control algorithm is introduced into the inverse Jacobian matrix control algorithm, with a control amount of


(23) $$\begin{array}{l} u_{h}\left(n\right)=\Delta f^{h}\left(n+1\right)=f^{h}\left(n+1\right)-f^{h}\left(n\right) \end{array}$$

In order to reduce the impact of system image processing time delay on the system, the compensation amount will be predicted ξ bringing it into the control algorithm to obtain the final visual servo control algorithm:


(24) $$\begin{array}{l} u_{h}\left(n+1\right)=J\left(K_{P}e_{h}\left(n\right)+K_{I}\overset{n}{\underset{i=0}{\sum }}e_{h}\left(n\right)\right)+kV_{image} \end{array}$$

**Figure 4:**
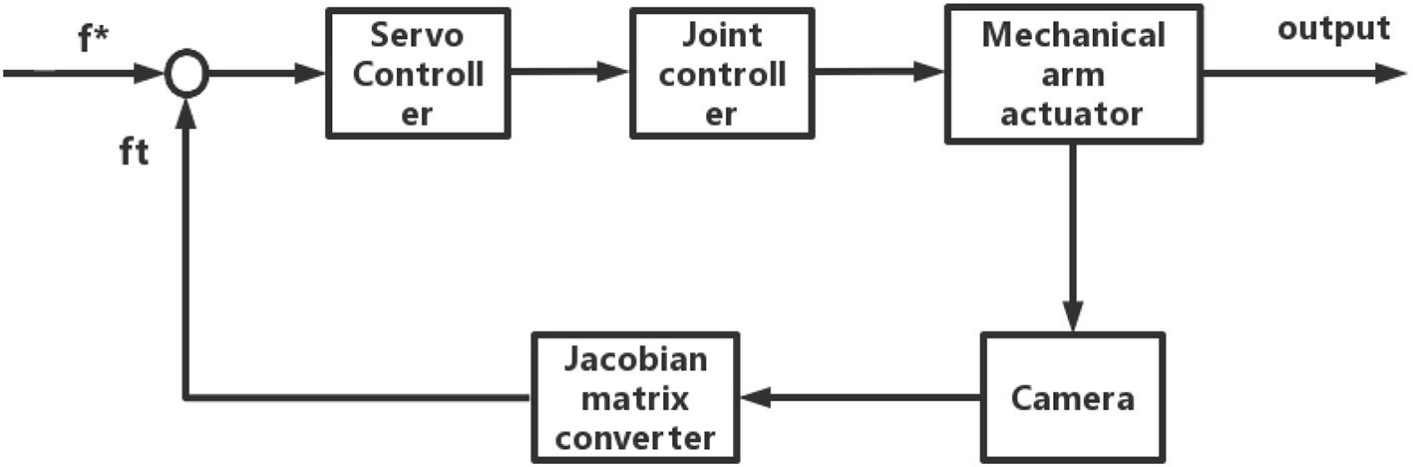
Framework diagram of the robot servo system.

*K*_*P*_ and *K*_*I*_ represent the proportional and differential coefficients, while k represents the predictive compensation coefficient of the system, which is related to the rate of change of image features. As shown in Figure 4, the robot control system is combined with the visual system to form a closed-loop robot visual servo system.

## 3. Experimental results

### 3.1. Simulation test

To verify the correctness of the uncalibrated visual servo algorithm, a robotic arm model, a monocular camera model, and a target object model were established in the simulation platform MATLAB by simulating real robotic arm servo experiments. A camera robotic arm model with “eyes in hand” was adopted, and the Jacobian online estimation algorithm using the dynamic quasi-Newton method was used for visual feedback. By using a visual controller, the control amount is calculated using image feature deviation to drive the end of the robotic arm to move toward the target. Finally, the effectiveness of the uncalibrated visual servo algorithm was verified through simulation experiments, providing a theoretical basis for practical development work.

We established a robotic arm model, monocular camera model, and target object model in the simulation platform MATLAB. The robotic arm is a six-axis Puma560 robotic arm. The camera has a resolution of 1,024 ^*^ 1,024, a focal length of 8 mm, and is installed at the end of the robotic arm (eye in hand). The target object is three small balls located above the robotic arm.

**Figure 5:**
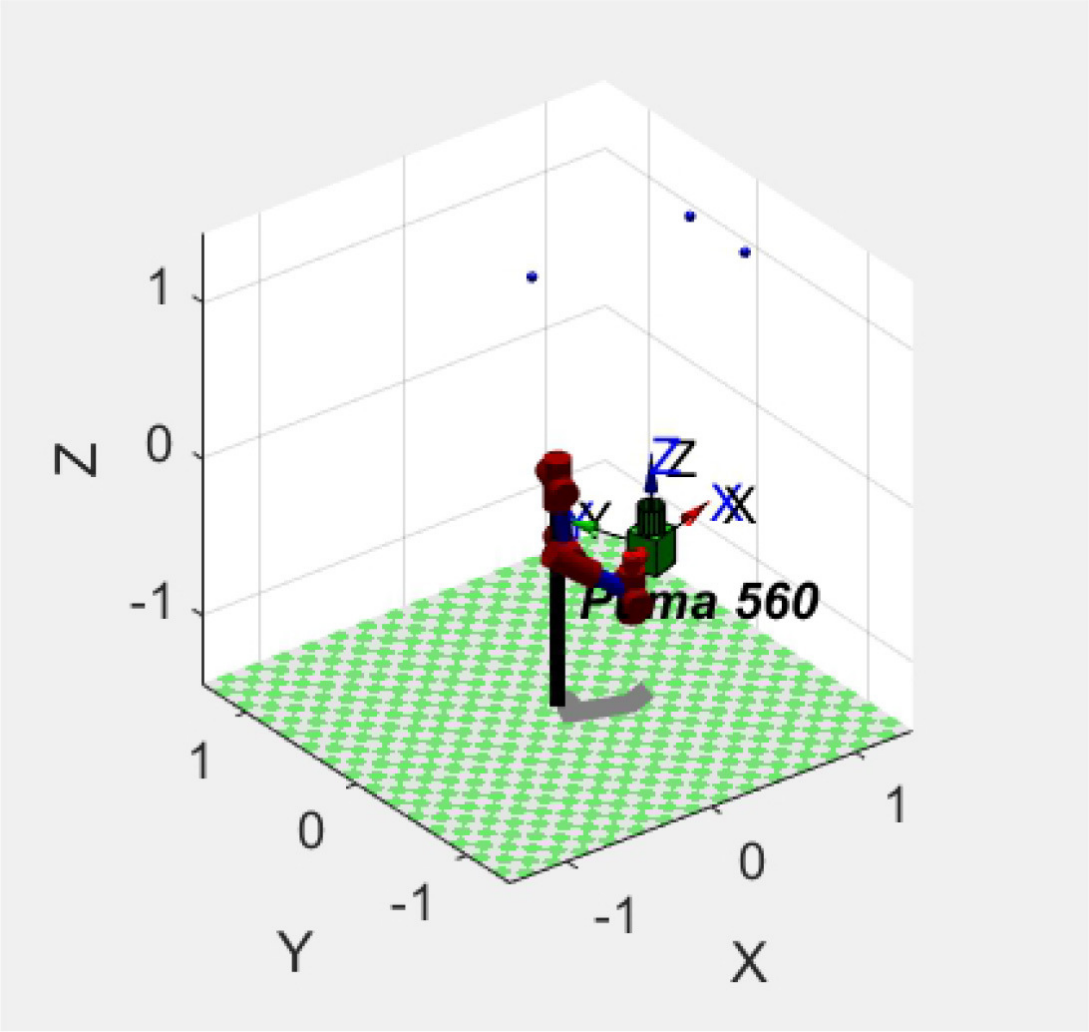
Simulation model of Puma560 Robot Arm Servo System.

At the initial moment, the end of the robotic arm undergoes six exploratory movements. As shown in Figure 5, it is a simulation model of the servo system. The robotic arm is Puma560, and the camera is installed at the end of the robotic arm in green. The three blue balls in the picture are the target objects. Robot movement generates displacement Δ*P*_0_ at the end of the robotic arm and the displacement of feature points Δ*F*_0_ within the image plane. The initial value of the Jacobian matrix is


(25) $$\begin{array}{l} J_{\text{0}}=\Delta F_{0}\cdot \Delta {P_{\text{0}}}^{\text{-1}} \end{array}$$

Using the dynamic quasi-Newton method to update the Jacobian matrix, the update frequency of the robotic arm is set to 0.1–0.2 mm per movement until the pixel error of the image reaches the range.

### 3.2. Experimental results of the dynamic quasi-Newton method

The error of the robotic arm in this experiment after 20 iterations is 0.31. After 35 iterations, the error is 0.011. After 56 iterations, the error was 0.0001, and the final image coordinates of the small ball were 761.999661.999, 761.999412.0, and 212.0661.999, respectively. The initial expected pixel coordinates were 762662, 762412, and 212662.

**Figure 6:**
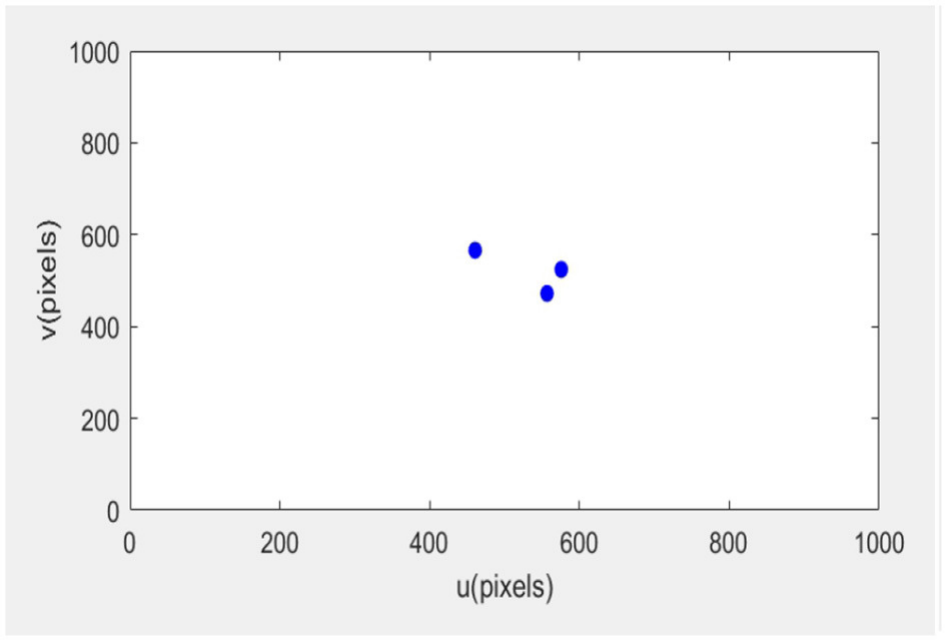
Initial position of the servo system.

**Figure 7:**
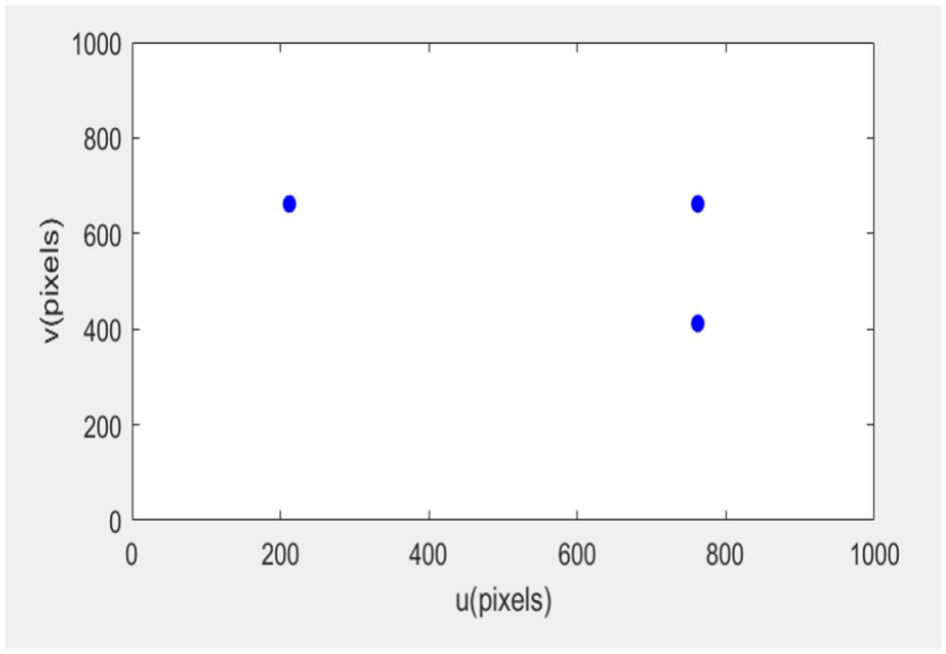
Position of the servo system in the end.

**Figure 8:**
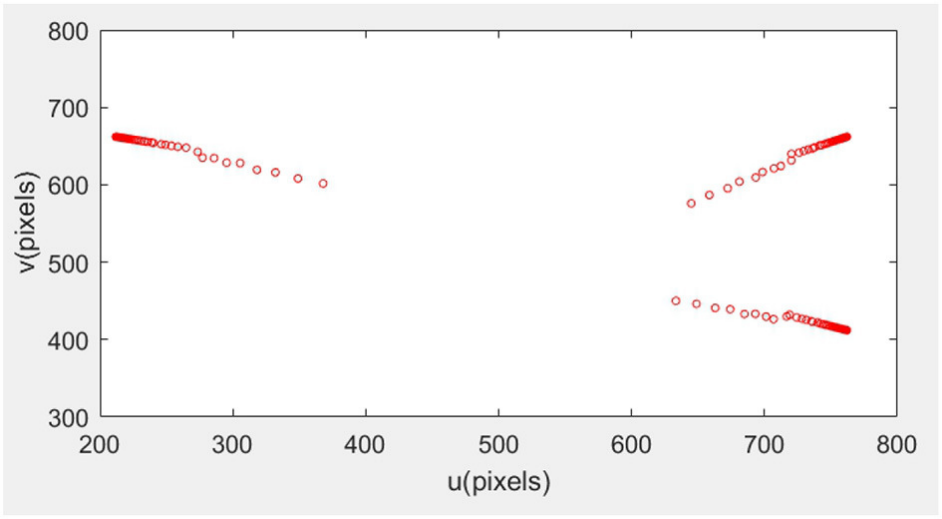
Motion trajectory of feature points in the image plane.

**Figure 9:**
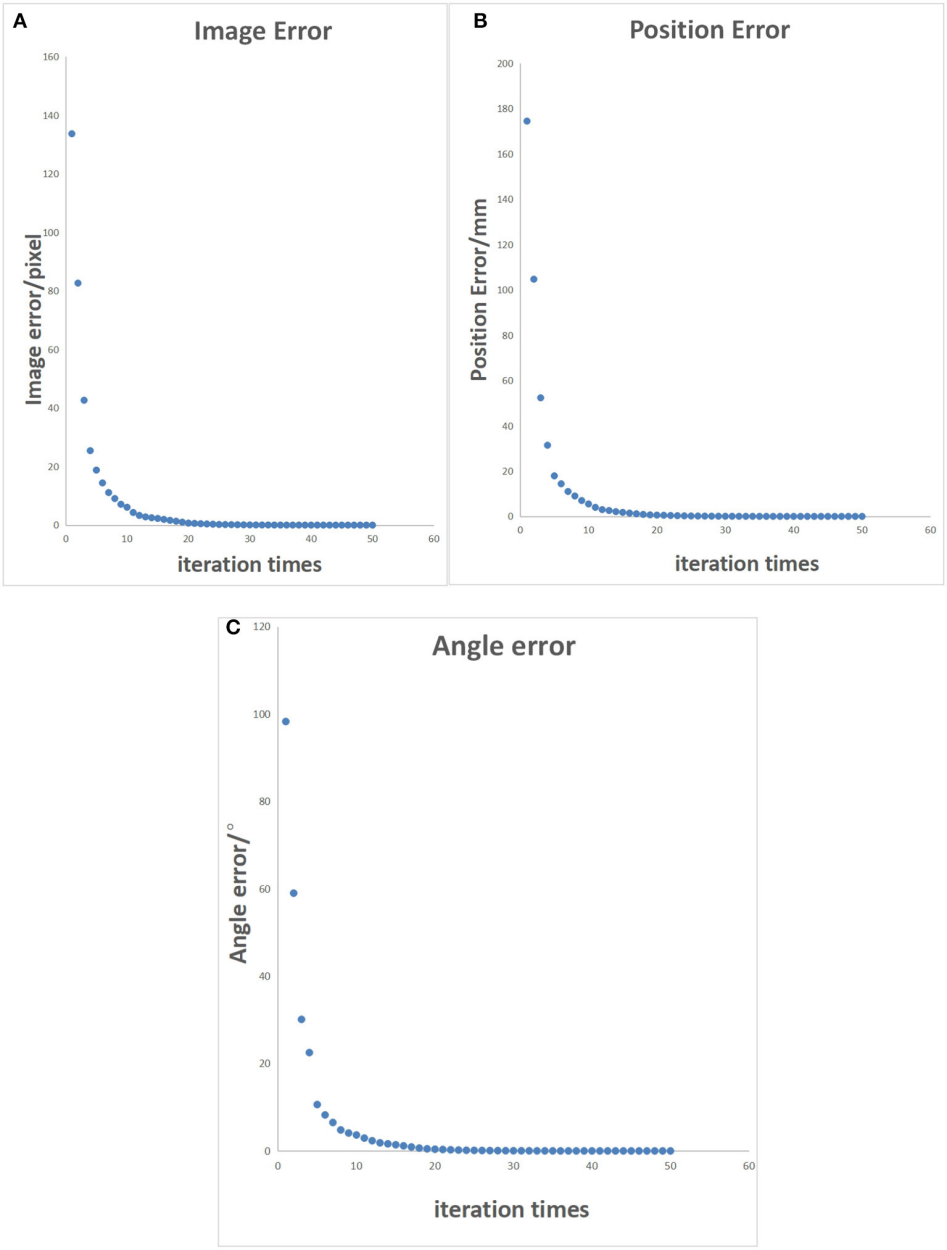
Characteristic points error control chart. **(A)** Pixel error of characteristic points on the image. **(B)** Position error of characteristic points in reality. **(C)** Angle error of the robotic arm.

The initial posture of the robotic arm servo system and the pixel coordinates of three small balls are shown in Figure 6. The posture and ball pixel coordinates at the end of the servo are shown in Figure 7. The motion trajectories of the feature points of three small balls in the image plane are shown in Figure 8. The error of the entire process (image error and robotic arm end pose error) varies with the number of cycles, as shown in Figure 9.

### 3.3. Orthogonal initialization method test results

According to the simulation experiment results shown in Table 1, it can be seen that the uncalibrated system requires multiple iterations to achieve the specified accuracy. However, in actual production environments, there is no enough time for iterative optimization. Looking at the simulation results data, it was found that the Jacobian matrix obtained from the uncalibrated initialization of the original scheme had a significant error in conversion. Through analysis, it was found that during the initialization process, images before and after movement were obtained by moving the robotic arm. In this process, there is coupling in the movement of the manipulator, which will lead to an irreversible and unsolvable Jacobian matrix.

**Table 1:** Characteristic point iteration error data table.

**Iterations**	**Image error/pixel**	**Position error/mm**	**Angle error/°**
1	133.68860	174.48133	98.2602
5	18.79910	17.89122	10.6121
10	6.10400	5.50026	3.6511
15	2.29155	1.76988	1.4074
20	0.70234	0.57714	0.3932
25	0.23001	0.18867	0.1286
30	0.07536	0.06163	0.0420
35	0.02469	0.02002	0.0137
40	0.00809	0.00639	0.0044
45	0.00265	0.00193	0.0014
50	0.00087	0.00047	0.0004

**Figure 10:**
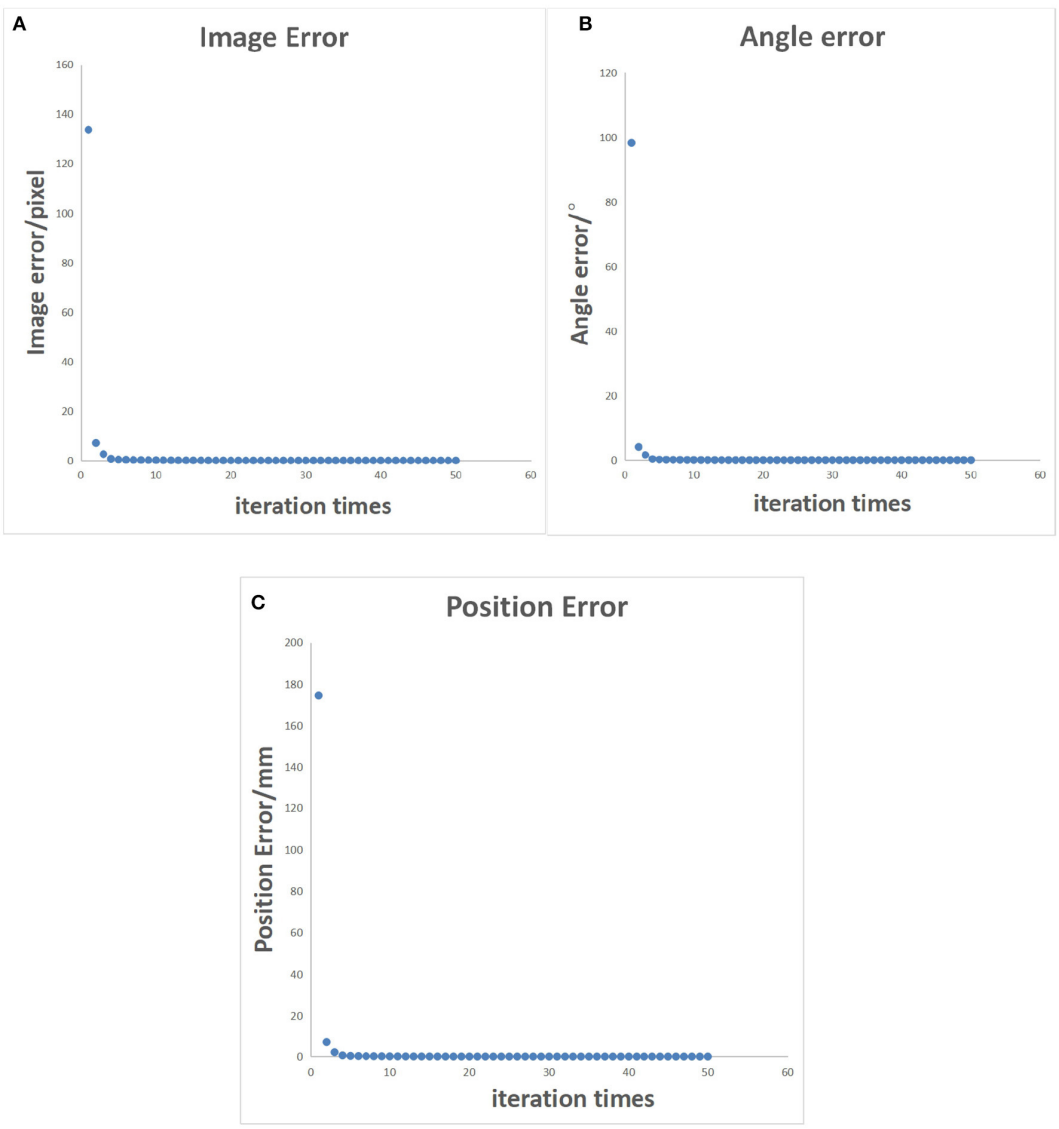
Error control chart after decoupled optimization. **(A)** Pixel error of characteristic points on the image. **(B)** Position error of characteristic points in reality. **(C)** Angle error of the robotic arm.

**Figure 11:**
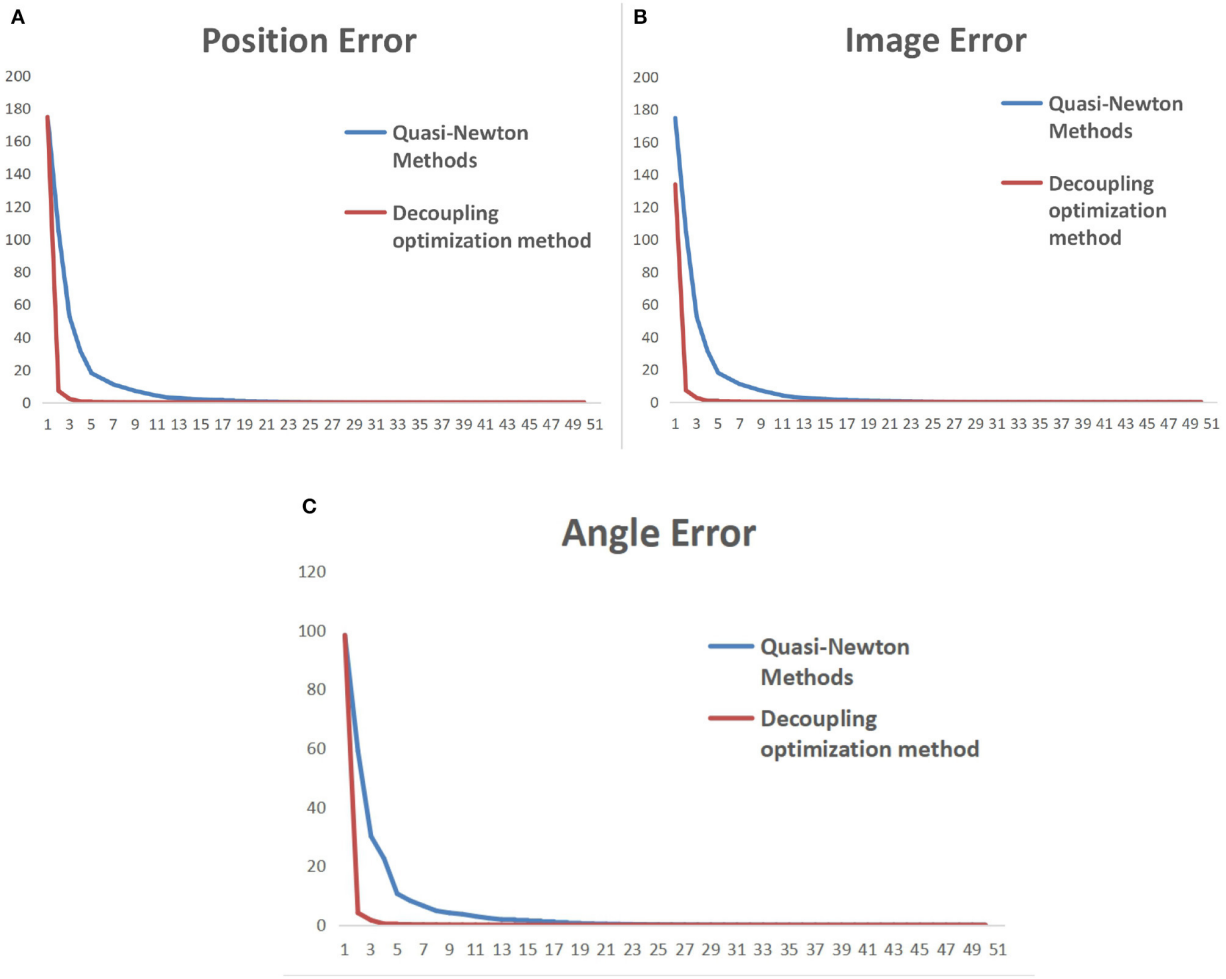
Comparison of iterative convergence before and after initialization optimization. **(A)** Pixel error of characteristic points on the image. **(B)** Position error of characteristic points in reality. **(C)** Angle error of the robotic arm.

To make the multiple sets of image feature points obtained after the robotic arm moves linearly uncorrelated, it is necessary to decouple the collected feature point set. This will be an incredibly complex and cumbersome task. Therefore, by standardizing the movement direction of the robotic arm during the initialization process, the obtained feature point set is naturally linearly uncorrelated. The iterative process error data of the uncalibrated system after the decoupling optimization initialization process is shown in Table 2, and the error of the entire process varies with the number of cycles, as shown in Figure 10. In Figure 11, it can be seen that the iterative speed of the Jacobian matrix after decoupling optimization has been significantly improved. Faster iterative convergence speed can effectively improve the real-time performance of robot visual servo systems during dynamic tracking.

**Table 2:** Optimized feature point iteration error data table.

**Iterations**	**Image error/pixel**	**Position error/mm**	**Angle error/°**
1	133.68860	174.48133	98.2602
2	7.1235	7	4.1
3	2.52	2.1	1.62
4	0.70234	0.57714	0.3932
5	0.4	0.32	0.2
25	0.0043	0.0037	0.0018
50	0.00087	0.00047	0.0004

In Figure 11, the vertical axis represents the error during the robot iteration process, and the horizontal axis represents the number of iterations. The update cycle for each iteration of the robot is not fixed. The iterative process includes camera shooting, image processing, and robot motion. Due to the different amount of information in each cycle, the iteration period will fluctuate between 20 and 30 ms.

### 3.4. Comparison of experimental results

**Figure 12:**
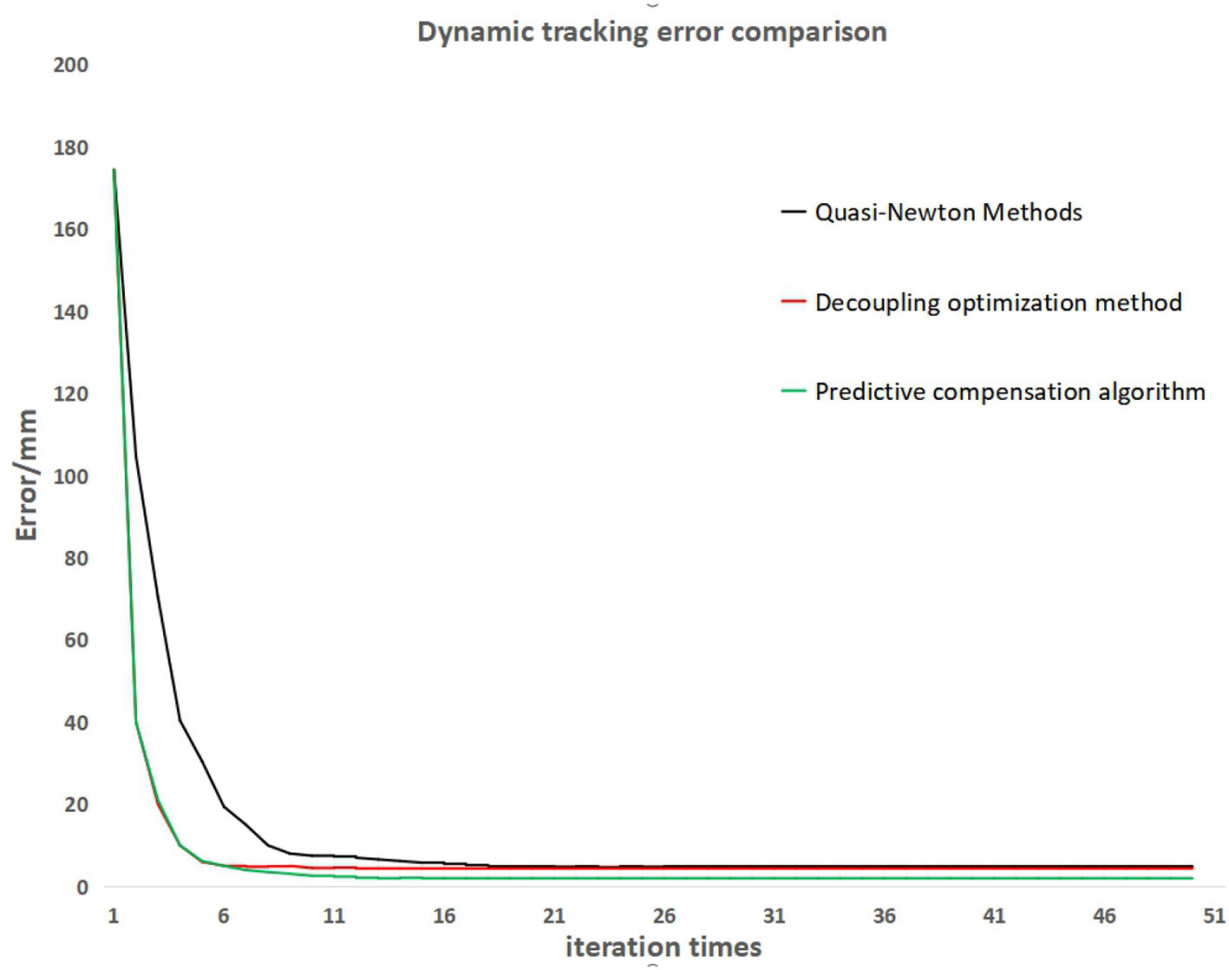
Dynamic tracking error comparison.

The above simulation tests have verified the reliability of the dynamic quasi-Newton method and the iterative algorithm after decoupling optimization. Next, the two algorithms mentioned above and the servo control algorithm with predictive compensation will be tested on the robotic arm. During the testing process, the robot dynamically tracks the target ball moving on the conveyor belt. The tracking process error data is recorded by identifying the distance between the centroid position of the target ball in the photos captured by the camera during the tracking process and the laser point position vertically shot by the robot arm. The tracking error curves of the three algorithms are shown in Figure 12.

From the tracking error curve in Figure 12, it can be observed that the iterative algorithm after decoupling optimization and the servo control algorithm with predictive compensation have a faster convergence speed than the dynamic equal Newton method. The servo control algorithm with predictive compensation can further reduce the tracking error in the convergence state.

## 4. Conclusion

This study investigates the application of the dynamic equal Newton method, the iterative algorithm after decoupling optimization, and the servo control algorithm with predictive compensation in robot uncalibrated visual servo systems. However, due to the dynamic equal Newton method requiring multiple iterations to obtain an accurate Jacobian matrix, a decoupling optimization method for the initialization process was proposed by analyzing the entire process of the uncalibrated robot visual servo system. The iterative algorithm after decoupling optimization can effectively reduce the number of iterations and improve the convergence speed of the Jacobian matrix through simulation testing. Therefore, this algorithm has a high practical value in production applications.

Due to the time lag that cannot be completely eliminated when moving from the visual system to the robot's active position information in the eye-in-hand mode, this study proposes a method called the servo control algorithm with predictive compensation to weaken or even eliminate the tracking error caused by the time lag. It showed a very significant effect on the experimental test results.

## Data availability statement

The original contributions presented in the study are included in the article/supplementary material, further inquiries can be directed to the corresponding author.

## Author contributions

HQ conducted theoretical research, algorithm design, and paper writing for the article. DH conducted simulation testing and built the framework of the robot visual servo system. BZ completed the collection and analysis of experimental data. MW has completed the optimization and revision of the paper content. All authors contributed to the article and approved the submitted version.
